# Modified Borg Scale (mBorg), the Numerical Rating Scale (NRS), and the Dyspnea- 12 Scale (D- 12): cross-scale comparison assessing the development of dyspnea in early-stage lung cancer patients

**DOI:** 10.1007/s00520-025-09474-x

**Published:** 2025-05-02

**Authors:** Hammoda Abu-Odah, Xian-Liang Liu, Tao Wang, Ivy Y. Zhao, Janelle Yorke, Jing-Yu Benjamin Tan, Alex Molassiotis

**Affiliations:** 1 School of Nursing, The Hong Kong Polytechnic University, Kowloon, Hong Kong SAR China; 2 School of Nursing and Health Studies, Hong Kong Metropolitan University, Homantin, Kowloon, Hong Kong SAR China; 3 Faculty of Health, Charles Darwin University, Brisbane Centre, Brisbane, QLD 4000 Australia; 4 School of Nursing and Midwifery, University of Southern Queensland, Ipswich, QLD 4305 Australia; 5 Centre for Health Research, University of Southern Queensland, Springfield, QLD 4300 Australia; 6 Division of Nursing, Midwifery and Social Work, University of Manchester, Manchester, UK; 7 College of Arts, Humanities and Education, University of Derby, Derby, UK

**Keywords:** Dyspnea, Dyspnea- 12 Scale, Early stage, Lung cancer, Modified Borg Scale, Numerical Rating Scale

## Abstract

**Background and objective:**

Assessing breathlessness in early-stage lung cancer has been complicated by using different rating scales, potentially leading to overestimation or underestimation of the experience. This study aims to examine the interscale concordance among three frequently used scales, the Modified Borg Scale (mBorg), the Numerical Rating Scale (NRS), and the Dyspnea-12 scale (D-12) and identify common factors contributing to breathlessness in post-operative early-stage lung cancer patients reported with refractory breathlessness.

**Study design and methods:**

A secondary analysis was conducted using the baseline data from a randomized controlled trial, focusing on 142 early-stage lung cancer patients. Breathlessness was evaluated using mBorg, NRS, and D-12 scales. Generalized linear regression explored relationships across scale ratings and identified factors associated with dyspnea.

**Results:**

The mean score on the mBorg scale was 4.28 ± 1.57 (range = 0–8), the NRS yielded a mean score of 4.73 ± 1.99 (range = 1–10), and the D-12 was 7.04 ± 2.88 (range = 2–17). This study revealed strong correlations among the mBorg, physical domain of D-12, and NRS scales (*r* = 0.67, *p* < 0.000), indicating that these measures yielded similar results in assessing the physical aspects of breathlessness. D-12 Total, and D-12 physical scores correlated highly with quality of life, while the D-12 emotional subscale showed weak correlations. Asthma and insomnia emerged as significant risk factors across all scales.

**Conclusion:**

This study highlights interscale concordance and key contributors to breathlessness in operable early-stage lung cancer patients. All three scales validly measure dyspnea, with the D-12 and NRS offering a holistic assessment by including affective-dyspnea scores.

## Introduction

Lung cancer is one of the most frequently diagnosed cancers and the leading cause of cancer-related deaths globally, with around 2.21 million people dying from the disease in 2020 [1]. Lung cancer is often not diagnosed until it has reached an advanced stage, with over 75% of cases being diagnosed at stage III or IV disease, significantly impacting survival rates [2]. Globally, the 5-year survival rate of lung cancer is 17.9% [3], while in China it is notably higher at 37.0% [4].

A critical challenge in improving these survival rates is the frequent under-detection of early symptoms, such as breathlessness, which can delay diagnosis and treatment. Breathlessness is often overlooked or misattributed to other conditions, leading to a delay in recognizing lung cancer. Ferreira et al. [5] highlight the issue of undetected dyspnea, emphasizing the need for improved assessment methods. The assessment of initial respiratory symptoms, which may signal lung cancer incidence, has sparked increased interest in order to identify lung cancer at an earlier stage [6]. Chronic breathlessness (dyspnea) is a common and distressing respiratory symptom experienced by people with early-stage lung cancer [7]. It is defined as a subjective experience of breathing discomfort [8] that persists despite optimal treatment of the underlying pathology, resulting in disability [9, 10]. Breathlessness is a highly subjective symptom, and the severity varies as it may be associated with the patient’s physiological, psychological, social, and environmental factors [11]. The negative impacts of breathlessness on patients with early-stage lung cancer have been clearly documented, with evidence showing its adverse effects on quality of life, sleep, fatigue, anxiety, and depression [7]. Measuring these symptoms can help demonstrate effective clinical strategies, assess the impact of interventions, and ultimately improve health outcomes for these patients. Thus, the assessment of breathlessness should be multidimensional, using patient-reported outcome measures that encompass its various dimensions in order to be able to evaluate the effects of treatment [12].

The assessment of breathlessness can be applied to examine patients’ breathlessness when doing exercise, and in daily life [13]. Commonly used assessment tools for breathlessness of lung cancer patients include the modified Borg scale (mBorg), Numerical Rating Scale (NRS) [14], and the Dyspnea- 12 (D- 12) questionnaire [15]. Several limitations are encountered in the assessment of breathlessness; one of these is about the choice of the most appropriate scale. The unidimensional nature of different scales requires the use of multiple instruments to gain a more systematic understanding of a patient’s breathlessness [15]. However, comparing current studies using these tools is challenging due to the measurement of different endpoints of breathlessness. The lack of cross-scale comparisons across mBorg, NRS, and D- 12 further complicates the ability to make meaningful comparisons and draw conclusions from the literature.

This study aimed to examine the interscale concordance among three frequently used scales, the mBorg, the NRS, and the D- 12, and identifying the primary sociodemographic and clinical factors contributing to breathlessness, as depicted by each scale, in operable early-stage lung cancer patients who experience chronic breathlessness. It was also aimed at exploring domains more prominently linked with dyspnea in each scale (i.e. physical function; emotional aspects; quality of life, etc.). By achieving the study’s objectives, this analysis can offer a more comprehensive insight into the measurement of breathlessness in early-stage lung cancer patients and identify those at a heightened risk of developing breathlessness.

## Methods

### Study design

This was a secondary analysis of a subset of baseline data from a randomized controlled trial [The clinical trial registration number (NCT03834116)] that examined the effectiveness of resistance inspiratory muscle training (IMT) in managing breathlessness in Chinese patients with thoracic malignancies]. Ethical approval was granted by the ethics committee at the Hong Kong Polytechnic University (HSEARS20180509003), the Human Research Ethics Committee at Charles Darwin University (H19013), and the Affiliated Hospital of Southwest Medical University (KY2018002).

### Setting

Participants were recruited from the Affiliated Hospital of Southwest Medical University in Mainland China.

### Selection criteria

The participants included in this study were early-stage lung cancer patients and were selected from the RCT main dataset using the following selection criteria: (i) diagnosed with stage I, IIA, or IIB lung cancer; (ii) adults aged 18 or above; (iii) expected prognosis of over 3 months as determined by the clinicians; (iv) oxygen saturation levels of 85% or higher while at rest; (v) confirmed as early-stage cancer patients experiencing breathlessness; and (vi) had refractory breathlessness that is unresponsive to the current treatment plan for the past two weeks [daily breathlessness for 3 months at rest or with minimal exertion despite receiving maximum treatment for contributing factors]. These individuals (*n* = 142) were selected from the baseline data set (*N* = 196) of the RCT parent study and included in the analysis for this study.

Per the RCT’s exclusion criteria, patients with unstable COPD characterized by frequent or acute exacerbations, rapidly deteriorating breathlessness necessitating urgent medical intervention, undergoing palliative radiotherapy to the chest within 4 weeks, or chemotherapy within 2 weeks, as well as those with intractable cough, unstable angina, or clinically diagnosed pleural effusion requiring drainage, were excluded from the study.

### Study procedures of the parent RCT study

All eligible patients with lung cancer who had undergone surgery who visited the outpatient clinic of the Affiliated Hospital of Southwest Medical University for treatment or follow-up were referred to the research team and invited to participate in the study. Those who agreed to participate were provided with full explanations of the study’s aims, significance, and consent procedures, participant rights of withdrawal, and details of participation. Participants were also provided with a detailed study information sheet. Patients were informed of the voluntary nature of participation and their right to withdraw at any time without prejudice. All participants signed a consent form prior to participation.

### RCT baseline data utilized in this study

#### Demographic and clinical characteristics

Demographic and clinical variables utilized in this secondary analysis include age, Body Mass Index (BMI), marital status, education, occupation, smoking and drinking habits, household income, surgery type, allergic history, hypertension, diabetes, heart diseases, asthma, pneumonectomies/emphysema, pulmonary tuberculosis, and insomnia.

#### Breathlessness

Three instruments were utilized to assess participants’ breathlessness levels, including the Modified Borg Scale (mBorg), Numerical Rating Scale (NRS), and Dyspnea- 12 questionnaire (D- 12). The mBorg is a vertical numeric scale, ranging from 0 “no perceived exertion/breathlessness” to 10 “maximal exertion/breathlessness” [16]. In this secondary analysis, the mBorg scores were divided into four categories: 0–0.5 = no breathlessness, 1–2 = mild breathlessness, 3–4 = moderate breathlessness, and ≥ 5 = severe breathlessness.

The NRS has been recommended for assessing the perceived severity of breathlessness (average and “worst” over the past 24 h, and “now”) [17, 18]. The NRS is an 11-grade scale ranging from 0 “no breathlessness/no distress due to the breathlessness” to 10 “worst imaginable breathlessness/distress due to breathlessness”. In this secondary analysis, the NRS scores were divided into four categories: 0 = no breathlessness, 1–3 = mild breathlessness, 4–6 = moderate breathlessness, and ≥ 7 = severe breathlessness [19].

The third instrument, D- 12, is a 12-item scale encompassing two subscales: physical (7 items) and emotional (5 items) [20]. Each item has four answers: none (0), mild (1), moderate (2), or severe (3). The D- 12 total scores range from 0 to 36, with high scores indicating greater severity. D- 12 and its sub-scores demonstrated excellent reliability and validity in chronic obstructive pulmonary disease patients, with Cronbach’s alpha = 0.88 [21].

#### Psychological distress

The Hospital Anxiety and Depression Scale (HADS) assessed participants’ anxiety and depression status. It has been widely used among cancer patients [22]. It is a 14-item well-established scale encompassing two subscales: anxiety (7 items) and depression (7 items). Each item has a score ranging from 0 to 3. The HADS total score ranges from 0 to 42, with a higher score indicating a high level of psychological distress. HADS-Total scores > 15 indicate psychologically distressed patients. For subscales, each one is computed and determined under the following three categories: normal (score of 0–7), borderline (score of 8–10), and severe cases (score of 11–21) [23].

#### Quality of life

The St. George Respiratory Questionnaire (SGRQ), with 50 items, was adopted to assess the breathlessness-related QOL [24]. The SGRQ covers three domains, including impact, symptoms, and activity. Scores of the SGRQ and each domain range from 0 to 100, with a higher score reflecting poorer QOL. Although SGRQ is designed for patients with obstructive airway diseases, it was validated to measure QOL in LC participants in a previous pilot trial [25]*.*

#### Functional exercise capacity

The 6-min walk test (6MWT) was utilized to measure functional exercise capacity [26]. Participants were asked to walk from end to end at their self-selected pace along a 30-m line. They were instructed to cover as much distance as they could within 6 min. The distance covered was measured and classified into four stages based on the distance covered: stage 0 (≥ 350 m), stage 1 (250–350 m), stage 2 (150–250 m), and stage 3 (< 150 m). A higher stage indicated worse functional capacity [27].

### Statistical methods

Data were analyzed using the IBM SPSS (version 25.0). Descriptive statistics were used to summarize the participants’ characteristics, breathlessness levels, QOL, anxiety and depression, and functional exercise capacity. Univariate statistical tests, an independent samples *t* test, analysis of variance (ANOVA), and Pearson correlation were utilized to identify variables associated with breathlessness levels. All variables with a *p* value ≤ 0.25 in univariate analysis were selected for the generalized linear regression model. All statistical analyses were two-tailed, and *p* < 0.05 was considered statistically significant.

## Results

### Characteristics of study participants (N = 142)

A total of 142 early-stage lung cancer participants with breathlessness from the parent RCT dataset were included in this secondary analysis. A total of 140 participants (98.6%) were married, and the mean age of the participants was 58.70 (± 10.02 years, range = 30–82) years. Half of the participants (*n* = 70) were classified as overweight and obese based on BMI. Approximately 34.5% of participants had a past history of smoking, while 64.8% reported never having smoked. Most of the participants (*n* = 119, 83.3%) had a history of allergies. Around half of the participants (*n* = 67, 47.2%) underwent right upper or lower lobectomy. Frequent co-morbidities observed were hypertension (23.2%), diabetes mellitus (7.7%), heart diseases (7.7%), and tuberculosis (4.2%).

Participants reported slight impairment of QOL, with a SGRQ mean total score of 28.27 ± 1 1.65. The prevalence of clinically significant anxiety, depression, or both, as diagnosed with HADS-Total, was 7.0%, with a mean score of 4.48 ± 4.43 (range = 0–25). The majority of patients (*n* = 133, 94.3%) did not report any impairment of their functional capacity. The detailed characteristics of the study participants are summarized in Table 1.

**Table 1:** Characteristics of the participants (*N* = 142)

Variables	Mean ± SD; *n* (%)	Variables	Mean ± SD; *n* (%)
**Age in years**	58.70 ± 10.02	Exercise (h/week) *	
**Body Mass Index**	23.11 ± 2.77	0–2	111 (78.2)
**Blood oxygen level (%)**	97.85 ± 1.15	3–4	20 (14.1)
**Marital status**		5–6	8 (5.6)
Married	140 (98.6)	> 6	2 (1.4)
Not married	2 (1.4)	Surgery types*	
**Education**		Left upper lobectomy	39 (27.5)
No formal education	10 (7.0)	Left lower lobectomy	11 (7.7)
Primary school	34 (23.9)	Right upper lobectomy	42 (29.6)
Secondary school	49 (34.5)	Right lower lobectomy	25 (17.6)
High school/vocational school	18 (12.7)	Other	22 (15.5)
Junior college diploma	17 (12.0)	Hypertension*	
University degree or above	14 (9.9)	Yes	33 (23.2)
Occupation*		No	109 (76.8)
Professional and technical personnel	15 (10.6)	**Diabetes mellitus**	
Manual worker	34 (23.9)	Yes	11 (7.7)
No longer working	64 (45.1)	No	131 (92.3)
Clerical or administrative worker	9 (6.3)	Heart diseases*	
Other	19 (13.4)	Yes	11 (7.7)
Monthly income (RMB)*		No	130 (91.5)
< 3000	74 (52.1)	**Asthma**	
3000–6000	52 (36.6)	Yes	3 (2.1)
> 6000	10 (7.0)	No	139 (97.9)
**Body Mass Index**		**Pneumonectomies/emphysema**	
Underweight (below 18.5)	6 (4.2)	Yes	6 (4.2)
Healthy weight (18.5–22.9)	66 (46.5)	No	136 (95.8)
Overweight (23.0–24.9)	33 (23.2)	**Pulmonary tuberculosis**	
Obesity (25.0 and above)	37 (26.4)	Yes	6 (4.2)
**Smoking**		No	136 (95.8)
Never smoked	92 (64.8)	**Allergies history**	
Current smoking	1 (0.7)	Yes	119 (83.8)
Previous smoking	49 (34.5)	No	23 (16.2)
**Drinking habits**		**Insomnia**	
Yes	53 (37.3)	Yes	50 (35.2)
No	89 (62.7)	No	92 (64.8)
HADS total*	4.48 ± 4.43	**QOL**	
**6MWT**		SGRQ-Symptom (Range 0–100)	27.94 ± 13.56
Stage 0	79 (56.0)	SGRQ-Activity (Range 0–100)	44.89 ± 14.75
Stage 1	54 (38.3)	SGRQ-Impact (Range 0–100)	19.68 ± 12.02
Stage 2	6 (4.3)	SGRQ-Total (Range 0–100)	28.27 ± 11.65
Stage 3	1 (0.7)		

*QOL* quality of life, *RMB* renminbi, *SD* standard deviation, *SGRQ* St. George Respiratory Questionnaire, *6MWT* 6-min walk test

^a^Includes those who are single, widowed, or divorced

^b^Includes housewife, unemployed, and retired

^*^ Missing < 1%

### Dyspnea/Breathlessness status

Table 2 shows the level of breathlessness among study participants across the three different scales. Almost all of the participants (*n* = 139, 97.8%) had moderate to severe breathlessness as determined by the mBorg scale, with a total mean score of the mBorg scale being 4.28 ± 1.57 (range = 0–8), which was slightly high at the midpoint of 4. Concerning NRS, two thirds of participants (*n* = 101, 71.1%) endured moderate to severe breathlessness over the past 24 h, with a total score of 4.73 ± 1.99 (range = 1–10), while only a quarter of participants (*n* = 36, 25.1%) reported current moderate to severe breathlessness, with an overall score of 2.65 ± 1.26 (range = 0–7). A total of 111 participants (79.9%) had moderate to severe breathlessness as determined by the D- 12 scale, with an overall mean score of 7.04 ± 2.88 (range = 2–17), and it was 6.70 ± 2.46 (range = 2–13) and 0.30 ± 0.83 (range = 0–5) for the D- 12 physical and D- 12 emotional subscale, respectively (Table 2).

**Table 2:** Prevalence of dyspnea among participants

Instruments	Mean ± SD; range *n* (%)
**mBorg scale**	4.28 ± 1.57; 0–8
No breathlessness	1 (0.7%)
Mild breathlessness	2 (1.4%)
Moderate breathlessness	81 (57.0%)
Severe breathlessness	58 (40.8%)
**NRS (worst dyspnea over the past 24 h) hours)**	4.73 ± 1.99; 1–10
Mild breathlessness	41 (28.9%)
Moderate breathlessness	69 (48.6%)
Severe breathlessness	32 (22.5%)
**NRS (current dyspnea)**	2.65 ± 1.26; 0–7
No breathlessness	1 (0.7%)
Mild breathlessness	105 (73.9%)
Moderate breathlessness	35 (24.6%)
Severe breathlessness	1 (0.7%)
**D- 12 total (range 0–36)**	7.04 ± 2.88; 2–17
No breathlessness	0 (0.0)
Mild breathlessness	29 (20.1%)
Moderate breathlessness	73 (52.5%)
Severe breathlessness	38 (27.4%)
D- 12 Physical	6.70 ± 2.46; 2–13
D- 12 Emotional	0.30 ± 0.83; 0–5

*D- 12* Dyspnea- 12 Scale, *mBorg* Modified Borg Scale, *NRS* Numerical Rating Scale, *SD* standard deviation

### Correlation across scales

Table 3 shows the correlation matrix among the scales. Pearson correlations indicated a moderately strong positive correlation between the mBorg and NRS scales (*r* = 0.67, *p* < 0.000), and a moderately positive correlation between mBorg and D- 12 (*r* = 0.49, *p* < 0.000). In addition, a moderately positive correlation between NRS and D- 12 was also reported (*r* = 0.54, *p* < 0.000). The findings also indicated a strong correlation among D- 12, its physical subscale, and QOL (*r* = 0.65, *p* < 0.000), as well as between D- 12 physical and mBorg, NRS, and QOL. Furthermore, moderate correlations were reported between the Total D- 12 scale and HADS (*r* = 0.37, *p* < 0.000).

**Table 3:** Pearson correlations (*r*) between dyspnea scales

	mBorg	NRS	6MWT	HADS	QOL (SGRQ)	D- 12	D- 12 Physical	D- 12 Emotional
mBorg	1							
NRS	0.67**	1						
6MWT	− 0.23**	− 0.24**	1					
HADS	0.09	0.13	0.01	1				
QOL (SGRQ)	0.60**	0.63**	− 0.39**	0.21*	1			
D- 12	0.49**	0.54**	− 0.27**	0.37**	0.65**	1		
D- 12 Physical	0.53**	0.60**	− 0.27**	0.29**	0.65**	0.96**	1	
D- 12 Emotional	0.10	0.70	− 0.12	0.42**	0.32**	0.60**	0.37**	1

*D- 12* Dyspnea- 12, *mBorg* Modified Borg Scale, *6MWT* 6-min walk test, NRS Numerical Rating Scale

^*^p < 0.05

^**^p < 0.01

However, no correlation was observed among D- 12 emotional and mBorg and NRS scales. There was a low correlation among 6MWT test and all mBorg, NRS, QOL, D- 12, and D- 12 physical scales.

### Risk factors associated with breathlessness

Univariate analysis of sociodemographic and clinical factors that influence breathlessness was applied to identify the potential factors of breathlessness across scales (Table 4). Variables with *p* values less than 0.25 in the univariate analysis were considered in the regression analysis. Further, generalized linear regression analyses were used to predict variables associated with breathlessness (Table 5). For the mBorg scale, findings revealed that insomnia (*β* = 0.788; *p* = 0.022), diabetes mellitus (DM) (*β* = 0.764; *p* = 0.040), and asthma (*β* = –1.870; *p* = 0.044) were risk factors for breathlessness among early-stage LC patients. In the D- 12 scale, findings also underscored that insomnia (*β* = 0.954; *p* = 0.015) and asthma (*β* = − 1.877; *p* = 0.000) were independently associated with breathlessness among LC patients. For the NRS, insomnia (*β* = 0.954; *p* = 0.015) and asthma (*β* = − 1.877; *p* = 0.009) were also independently associated with breathlessness among early-stage LC patients. In conclusion, asthma and insomnia, which were shared variables across the three scales, were strongly considered the main risk factors associated with breathlessness among early-stage LC patients (Table 5).

**Table 4:** Univariate analysis of sociodemographic and clinical factors that influence dyspnea

Variables	mBorg scale		NRS scale		D- 12 scale	
	Mean (SD)	*p* value	Mean (SD)	*p* value	Mean (SD)	*p* value
**Age**		0.586		0.634		0.476
≤ 44 years	4.00 (2.04)		4.45 (2.46)		6.18 (2.63)	
45–64 years	4.39 (1.52)		4.86 (2.04)		7.23 (3.12)	
≥ 65 years	4.15 (1.56)		4.56 (1.79)		6.88 (2.42)	
**Marital status**		0.412		0.386		0.717
Married	4.30 (1.58)		4.75 (2.00)		7.05 (2.89)	
Not married	3.00 (0.00)		3.00 (0.00)		6.00 (0.00)	
**Education**		0.528		0.372		0.544
No formal education	4.80 (1.47)		5.40 (1.57)		8.40 (3.43)	
Primary school	4.61 (1.47)		4.85 (2.04)		7.20 (3.04)	
Secondary school	4.20 (1.68)		4.84 (2.20)		7.04 (2.92)	
High school/vocational school	4.05 (1.39)		3.83 (1.68)		6.76 (3.01)	
College diploma	4.17 (1.42)		4.94 (1.24)		7.00 (2.54)	
University degree or above	3.85 (1.91)		4.50 (2.37)		6.07 (2.09)	
**Occupation**		**0.007**		**0.000**		0.429
Professional and technical personnel	4.53 (1.72)		5.20 (1.89)		6.80 (2.54)	
Manual worker	4.88 (1.59)		5.47 (1.74)		7.73 (3.10)	
No longer working	4.12 (1.55)		4.72 (1.87)		7.04 (2.99)	
Clerical or administrative worker	4.88 (1.83)		5.00 (2.44)		6.37 (2.87)	
Other	3.36 (0.76)		3.05 (1.87)		6.26 (2.35)	
**Monthly Income (RMB)**		**0.027**		0.885		0.414
< 3000	4.64 (1.62)		4.88 (2.06)		7.40 (2.89)	
3000–6000	3.94 (1.37)		4.75 (1.97)		6.74 (2.95)	
> 6000	3.80 (1.98)		4.60 (1.43)		6.70 (2.71)	
**Body Mass Index**		0.093		0.258		0.987
Underweight (below 18.5)	5.83 (2.13)		5.83 (1.60)		7.33 (1.75)	
Healthy weight (18.5–22.9)	4.13 (1.47)		4.58 (2.01)		7.06 (2.83)	
Overweight (23.0–24.9)	4.33 (1.70)		5.12 (2.17)		7.09 (2.95)	
Obesity (25.0 and above)	4.27 (1.57)		4.49 (1.80)		6.91 (3.13)	
**Smoking**		0.164		0.229		0.206
Never smoked	4.34 (1.62)		4.77 (2.01)		7.31 (3.07)	
Current smoking	7.00 (0.00)		8.00 (0.00)		9.00 (0.00)	
Previous smoking	4.12 (1.46)		4.59 (1.94)		6.46 (2.41)	
**Drinking habits**		0.801		0.944		0.066
Yes	4.24 (1.61)		4.72 (2.04)		6.46 (2.42)	
No	4.31 (1.56)		4.74 (1.98)		7.39 (3.08)	
**Exercise (h/week)**		0.653		0.482		0.560
0–2	4.35 (1.55)		4.61 (1.99)		7.21 (2.91)	
3–4	4.20 (1.60)		5.15 (2.13)		6.50 (3.00)	
5–6	4.12 (2.03)		5.50 (1.92)		7.00 (2.16)	
> 6	3.00 (0.00)		5.00 (0.00)		5.00 (1.41)	
**Surgery types**		0.287		0.094		0.370
Left/Right upper lobectomy	4.43 (1.63)		4.96 (1.68)		6.90 (2.70)	
Left/Right lower lobectomy	4.83 (1.59)		5.61 (1.99)		7.40 (2.82)	
**Hypertension**		0.349		0.779		0.859
Yes	4.51 (1.69)		4.82 (2.24)		7.12 (3.27)	
No	4.22 (1.54)		4.71 (1.92)		7.01 (2.76)	
**Diabetes mellitus**		0.248		0.118		**0.025**
Yes	4.81 (1.72)		5.64 (1.96)		8.90 (3.93)	
No	4.24 (1.56)		4.66 (1.98)		6.88 (2.73)	
**Heart diseases**		0.653		0.253		0.478
Yes	4.09 (1.81)		4.09 (2.16)		6.45 (3.26)	
No	4.31 (1.56)		4.81 (1.97)		7.10 (2.86)	
**Asthma**		**0.000**		**0.001**		**0.050**
Yes	3.00 (0.00)		3.00 (0.00)		5.00 (1.00)	
No	4.31 (1.58)		4.77 (2.00)		7.08 (2.89)	
**Pneumonectomies/emphysema**		0.472		0.339		0.639
Yes	5.50 (1.87)		5.83 (1.94)		8.83 (2.48)	
No	4.23 (1.55)		4.68 (1.99)		6.96 (2.87)	
**Pulmonary tuberculosis**		0.472		0.480		0.639
Yes	3.83 (1.32)		4.17 (1.32)		6.50 (1.51)	
No	4.30 (1.58)		4.76 (2.02)		7.06 (2.92)	
**Allergies history**		0.234		0.437		0.691
Yes	4.34 (1.64)		4.79 (2.04)		7.08 (2.94)	
No	4.00 (1.16)		4.43 (1.75)		6.81 (2.53)	
**Insomnia**		**0.050**		0.106		**0.000**
Yes	4.64 (1.68)		5.10 (2.18)		8.62 (3.36)	
No	4.09 (1.49)		4.53 (1.86)		6.20 (2.18)	

The Bold data refer to the significant *p*- value at 0.05

*RMB* renminbi, *SD* standard deviation

**Table 5:** Generalized linear regression model for factors associated with dyspnea in the three scales

Scale	Variable	*β*	SE	95% CI	Wald	*p*
**mBorg scale**	Occupation					
	Professional and technical personnel	0.518	0.66	− 0.78–1.81	0.610	0.435
	Manual worker	0.740	0.57	− 0.39–1.87	1.650	0.199
	No longer worker	0.222	0.54	− 0.84–1.28	0.168	0.682
	Clerical or administrative worker	2.069	0.76	0.54–3.56	7.367	0.070
	Other	Ref	-	-	-	-
	Monthly income					
	< 3000	1.310	0.50	0.31–2.30	6.70	0.051
	3000–6000	0.296	0.50	− 0.70–1.29	0.33	0.866
	> 6000	Ref	-	-	-	-
	Body Mass Index					
	Underweight (below 18.5)	− 2.21	1.48	− 5.12–0.70	2.20	0.671
	Healthy weight (18.5–22.9)	− 3.38	1.40	− 6.12– − 0.61	5.74	0.393
	Overweight (23.0–24.9)	− 2.97	1.41	− 5.74– − 0.20	4.41	0.458
	Obesity (25.0 and above)	Ref	-	-	-	-
	Smoking					
	Current smoking	1.68	1.39	− 1.02–4.39	1.48	0.198
	Previous smoking	− 0.40	0.27	− 0.94–0.13	2.17	0.083
	Never smoked	Ref	-	-	-	-
	Diabetes mellitus [Yes]	0.764	0.49	− 0.19–1.72	2.42	**0.040**
	Asthma [Yes]	− 1.870	1.02	− 3.88–0.14	3.30	**0.044**
	Allergic history [Yes]	0.433	0.33	− 0.23–1.09	1.64	0.250
	Insomnia [Yes]	0.788	0.295	0.20–1.36	7.102	**0.022**
**NRS scale**	Occupation					
	Professional and technical personnel	0.226	0.609	− 0.73–1.65	0.578	0.768
	Manual worker	0.539	0.536	− 0.37–1.72	1.582	0.440
	No longer working	0.001	0.481	− 0.79–1.08	0.091	0.998
	Clerical or administrative worker	1.377	0.672	− 0.45–2.18	1.650	0.124
	Other	Ref	-	-	-	-
	Smoking					
	Current smoking	2.496	1.63	− 0.51–5.90	2.709	0.137
	Previous smoking	− 0.321	0.29	− 0.90–0.24	1.286	0.331
	Never smoked	Ref	-	-	-	-
	Surgery types					
	Left/Right upper lobectomy	− 0.347	0.341	− 1.01–0.322	1.03	0.309
	Left/Right lower lobectomy	Ref	-	-	-	-
	Diabetes Mellitus [Yes]	0.964	0.56	− 0.17–2.10	2.74	0.098
	Asthma [Yes]	− 1.877	0.96	− 3.76–0.01	3.79	**0.009**
	Insomnia [Yes]	0.954	0.30	0.35–1.55	9.756	**0.015**
**D- 12 scale**	Smoking					
	Current smoking	2.519	2.25	− 2.46–7.50	0.98	0.322
	Previous smoking	− 0.481	0.50	− 0.14–0.50	0.914	0.339
	Never smoked	Ref	-	-	-	-
	Drinking habits [Yes]	− 0.417	0.49	− 1.38–0.54	0.719	0.396
	Diabetes Mellitus [Yes]	1.861	0.80	0.28–3.44	5.325	**0.021**
	Asthma [Yes]	− 3.48	1.49	− 6.42– − 0.55	5.42	**0.020**
	Insomnia [Yes]	2.290	0.46	1.38–3.19	24.48	**0.001**

The Bold data refer to the significant *p*- value at 0.05

*CI* confidence interval, *D- 12* Dyspnea- 12 Scale, *mBorg* Modified Borg Scale, *NRS* Numerical Rating Scale, *SE* standard error

## Discussion

This study sheds light on early assessment and factors associated with breathlessness in operable early-stage lung cancer patients experiencing breathlessness using different scales. Our results show variations in the level of breathlessness among the study participants, depending on the scales used for assessment. Despite these variations, a strong positive correlation was observed among mBorg, NRS, and the D- 12 scales, indicating that these tools, while different, can yield comparable results in assessing breathlessness. Additionally, the study consistently found that asthma and insomnia were associated with breathlessness among early-stage lung cancer patients, highlighting their relevance across all three scales.

The presence of breathlessness in operable early-stage lung cancer patients has been found to have a detrimental impact on their overall health status [28–30]; however, it is concerning that breathlessness is frequently undetected and inadequately addressed in clinical practice [29, 31]. This may be attributed to the inaccurate estimation of the severity of breathlessness in this patient group. Our findings reported variation in the level of breathlessness among early-stage cancer patients following surgery, depending on the utilized scale. This highlights the variability in accurately assessing and quantifying the severity of breathlessness in our sample. The American Thoracic Society called for an appropriate measurement of the core domains of breathlessness [32]. Indeed, the choice of scale used for assessing breathlessness can significantly impact the estimation of its severity; thus, it is crucial to select an appropriate scale for a given sample.

In our study, we observed strong positive correlations among NRS, mBorg, and D- 12 scales, suggesting that these measures yielded similar results in assessing physical aspects of breathlessness. This is in line with the Borg Scale aim, that is used for assessing individual’s effort and exertion, breathlessness, and fatigue [16, 33]. Previous studies showed strong correlations between the NRS and Borg Scale [34]. Additionally, the D- 12 showed a stronger association with QOL and psychological distress, suggesting that the D- 12 scale may capture aspects of breathlessness that are closely related to overall well-being, and emotional aspects. The findings match with those reported by Tan et al., which confirmed the positive correlation between the D- 12 and HADS [15]. Considering these findings, it is crucial to adopt precise patient-reported outcome measures when assessing the level of breathlessness in routine lung cancer care. Using an appropriate scale will enable the identification of patients who need targeted cancer care for managing their breathlessness thereby reducing the burden in routine care. Thus, using multidimensional measures for evaluating breathlessness may be more accurate for capturing the level of breathlessness as well as providing a comprehensive evaluation of the impact of clinical interventions [35]. Further robust research is necessary to evaluate the accuracy of the aforementioned scales employed in assessing breathlessness in cancer patients. In addition, our results indicated that the 6MWT test had a low correlation with most scales, specifically, no significant correlation with the HADS and D- 12 emotional scales. This suggest that these scales may not effectively measure exercise endurance/walking capacity. These findings are in line with other literature that has reported a lack of correlation between the 6MWT and patient-reported outcome measures [36].

Despite the high correlation across mBorg, NRS, and D- 12, our findings further highlight the discrepancies in identifying the factors associated with breathlessness among lung cancer patients when utilizing different scales. Interestingly, among the shared variables across the three scales, only two factors, namely insomnia and asthma, were consistently associated with breathlessness among lung cancer patients. Breathlessness and insomnia in lung cancer patients are both interconnected factors and well-established in the medical literature for having a substantial effect on patient well-being [37]. Breathlessness can have a significant impact on cancer patient’s quality of sleep, effecting their overall sense of well-being [38]. Assessing and managing breathlessness effectively is essential to improve the quality of sleep for cancer patients. This includes identifying the underlying causes of breathlessness, such as tumor burden, treatment-related side effects, or comorbidities, and implementing appropriate interventions tailored to individual needs. This implies the importance of considering these specific factors when assessing and managing breathlessness in this patient population.

This study is one of the first to methodically reveal variations in the level of breathlessness among operable early-stage lung cancer participants, depending on the assessment scales used. It highlighted a strong positive correlation among mBorg, NRS, and the D- 12 scales, underscoring the need for healthcare providers to carefully select the appropriate scale for assessing breathlessness, as this choice can significantly influence clinical decision-making and patient management strategies. The findings showed that NRS and D- 12 reported a similar prevalence of moderate to severe dyspnea among participants when measuring the occurance of dyspnea in the prior 24 h. However, the results differed when assessing the current status of dyspnea using the NRS scale. Therefore, these two scales can significantly impact the estimation of breathlessness severity.

This study had two limitations. First, since study participants were postoperative patients with lung cancer, the study may understimate the current breathlessness experienced by preoperative patients. Second, the study participants were early stage, Chinese, and followed up at a single institution; the findings may not generalize beyond these sample characteristics. Future research should aim to include a more diverse patient population and consider longitudinal studies to better understand the progression of breathlessness over time.

## Conclusion

This study is among the first to assess interscale concordance and identify key contributors to breathlessness specifically in operable early-stage lung cancer patients. A strong positive correlation was found among the scores of the mBorg, NRS, and the D- 12 scales. Findings suggest that all three scales provide valid measurement of dyspnea in this participant group. However, the D- 12 and NRS scales also provide a score for affective-dyspnea, offering a more holistic assessment of breathlessness.

## Data Availability

The datasets generated and analyzed during the current study are not publicly available due to ethical reasons.
